# Cost-effectiveness of a national enterovirus 71 vaccination program in China

**DOI:** 10.1371/journal.pntd.0005899

**Published:** 2017-09-11

**Authors:** Wenjun Wang, Jianwen Song, Jingjing Wang, Yaping Li, Huiling Deng, Mei Li, Ning Gao, Song Zhai, Shuangsuo Dang, Xin Zhang, Xiaoli Jia

**Affiliations:** 1 Department of Infectious Diseases, The Second Affiliated Hospital of Xi’an Jiaotong University, Xi’an, China; 2 Department of Dermatology, Xi’an Children’s Hospital, Xi’an, China; 3 Department of Pediatrics, The Second Affiliated Hospital of Xi’an Jiaotong University, Xi’an, China; 4 The Second Department of Infectious Diseases, Xi’an Children’s Hospital, Xi’an, China; Georgia Southern University Jiann-Ping Hsu College of Public Health, UNITED STATES

## Abstract

**Background and aims:**

Enterovirus 71 (EV71) has caused great morbidity, mortality, and use of health service in children younger than five years in China. Vaccines against EV71 have been proved effective and safe by recent phase 3 trials and are now available in China. The purpose of this study was to evaluate the health impact and cost-effectiveness of a national EV71 vaccination program in China.

**Methods:**

Using Microsoft Excel, a decision model was built to calculate the net clinical and economic outcomes of EV71 vaccination compared with no EV71 vaccination in a birth cohort of 1,000,000 Chinese children followed for five years. Model parameters came from published epidemiology, clinical and cost data.

**Results:**

In the base-case, vaccination would annually avert 37,872 cases of hand, foot and mouth disease (HFMD), 2,629 herpangina cases, 72,900 outpatient visits, 6,363 admissions to hospital, 29 deaths, and 945 disability adjusted life years. The break-even price of the vaccine was $5.2/dose. When the price was less than $8.3 or $14.6/dose, the vaccination program would be highly cost-effective or cost-effective, respectively (incremental cost-effectiveness ratio less than or between one to three times China GDP per capita, respectively). In one-way sensitivity analyses, the HFMD incidence was the only influential parameter at the price of $5/dose.

**Conclusions:**

Within the price range of current routine vaccines paid by the government, a national EV71 vaccination program would be cost-saving or highly cost-effective to prevent EV71 related morbidity, mortality, and use of health service among children younger than five years in China. Policy makers should consider including EV71 vaccination as part of China’s routine childhood immunization schedule.

## Author summary

Enterovirus 71 (EV71) has caused great morbidity, mortality, and use of health service in children younger than five years in China. Recently, effective and safe vaccines against EV71 have been approved. Whether EV71 vaccination should be included as part of China’s routine childhood immunization schedule is unknown. In this study, we built a decision model to evaluate the health impact and cost-effectiveness of a national EV71 vaccination program in China. We find that vaccination would annually avert 567,500 cases of hand, foot and mouth disease (HFMD), 40,000 herpangina cases, 1,093,500 outpatient visits, 95,500 admissions to hospital, 435 deaths, and 14,000 disability adjusted life years based on the current Chinese birth cohort size. The break-even price of the vaccine was $5.2/dose. Within the price range of current routine vaccines paid by the government, a national EV71 vaccination program would be cost-saving or highly cost-effective. Policy makers should consider including EV71 vaccination as part of China’s routine childhood immunization schedule.

## Introduction

Enterovirus 71 (EV71) is one of the major agents that cause outbreaks of hand, foot, and mouth disease (HFMD) and herpangina worldwide[1]. Since the 1990s, the epidemic has mainly affected the Asia-Pacific region and EV71 has become a major public health issue across this region[2,3,4,5].

HFMD is characterized with fever and cutaneous lesions on hands, feet and buttocks, along with oral lesions. Although most cases are mild and self-limiting with an average duration of 7 days, approximately 1% can rapidly develop severe and even life-threatening complications such as encephalitis, aseptic meningitis, pulmonary oedema/hemorrhage and heart failure[1].

During the period from 2008 to 2012, China reported more than 7 million children with HFMD, of which around 45% were associated with EV71[6]. During the period from May 2008 to December 2014, China reported death of 2,225 children due to HFMD, with a case-fatality rate of 0.03% and 93% of them were associated with EV71 [6,7].

Current treatment is only to relieve symptoms. No specific drug to treat EV71 infection is available [1]. With limited impact of personal and environmental hygiene, vaccination is considered as the most effective and promising strategy to prevent HFMD and herpangina caused by EV71 [8].

Since 2013, three phase 3 randomized clinical trials (RCTs) to evaluate efficacy of inactivated EV71 vaccines in infants and young children have been completed in China [9,10,11]. The vaccines showed high efficacy and satisfactory safety to provide protection against EV71-associated diseases and are now available in China.

In 2010, before the key clinical trials were initiated, an cost-effectiveness analysis estimated economic value of a future vaccine against EV71[12]. Here, to assist policy makers in evaluating the implication of a national EV71 vaccination program in China, we reassessed the public health impact and cost-effectiveness of EV71 vaccination, using new evidence on the vaccine safety and efficacy as well as updated clinical and economic data on EV71 associated infections.

## Methods

### Model overview

**Fig 1:**
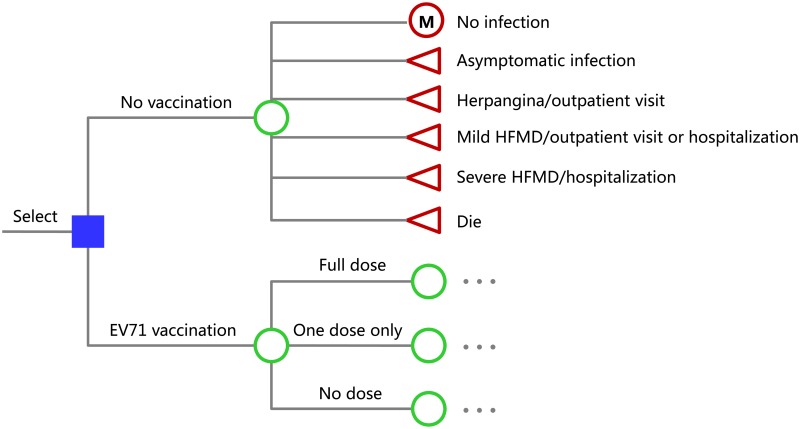
The decision model for implementation of an enterovirus 71 (EV71) immunization program in China. A birth cohort of 1,000,000 Chinese infants will receive the strategy of EV71 vaccination or no EV71 vaccination. In each strategy, they will annually experience one of the following conditions with certain probabilities: not infected with EV71, asymptomatic infection, herpangina, mild hand, foot and mouth disease (HFMD), severe HFMD, or death. For those not infected with EV71 in a year will experience one of the above conditions in the next year again (a Markov model showed as “M” in the figure). Each of the three branches rooted from EV71 vaccination (full dose, one dose only, and no dose) has the same branches as no vaccination (omitted in the figure).

Using Microsoft Excel, a decision tree model was built to calculate the net clinical and economic outcomes of EV71 vaccination compared with no EV71 vaccination (Fig 1). This model adopted Markov chain and hypothesized a 2012 birth cohort of 1,000,000 Chinese children. As most affected cases are younger than five years and the rates of severe illness and mortality decrease substantially in older children and adults[6], the model’s time horizon was five years after birth. The time step was one year. If children experienced symptomatic infection of EV71, they died or suffered from one of the following diseases: herpangina, mild HFMD, and severe HFMD[6]. Patients with HFMD were categorized as severe if they had any neurological complications (encephalitis, aseptic meningitis, or flaccid paralysis), or cardiopulmonary complications (pulmonary hemorrhage, pulmonary oedema, or myocarditis), or both; otherwise, they were classified as mild cases[6]. According to experience in China, almost all cases with HFMD make outpatient visits before deciding to receive home care or to be hospitalized for further treatment; a small number of cases with mild HFMD, almost all cases with severe HFMD and almost all death cases are hospitalized; herpangina alone is not an indication for hospitalization. Life years lost after the 5 years were taken into accounted. Accordingly, the model simulated events over a 5 year horizon but accounted for outcomes over the total lifetime of the simulated individuals. The primary result was presented as costs per disability adjusted life year (DALY) averted.

### Epidemiology

The overall incidence of HFMD was 1.2 per 1,000 person-years from 2008 to 2012, varied among provinces ranging from 0.2 in Tibet to 3.1 in Hainan according to the Chinese Center for Disease Control and Prevention (China CDC)[6,13]. EV71 accounted for 45% of mild, 80% of severe, and 93% of fatal cases and these proportions did not vary significantly with age among children aged 5 and under[6]. Thus, we calculated the annual incidences of mild, severe, and fatal EV71-associated HFMD by age from the corresponding overall incidences of HFMD by age and the proportions associated with EV71. We used their average values of four years (2009–2012) in our base-case analysis (Table 1).

**Table 1:** Parameters used in the cost-effectiveness analysis.

Parameter	Value	Range	Source
Incidence of HFMD, annually	1.2/1,000	0.2–3.1/1,000	[6]
Incidence of mild EV71-associated HFMD, annually			
< 12 months	6,048/million	-	[6]
12–23 months	12,773/million	-	[6]
24–59 months	7,527/million	-	[6]
Incidence of severe EV71-associated HFMD, annually			
< 12 months	186/million	-	[6]
12–23 months	381/million	-	[6]
24–59 months	129/million	-	[6]
Mortality of EV71-associated HFMD, annually			
< 12 months	8.3/million	-	[6]
12–23 months	13.3/million	-	[6]
24–59 months	3.5/million	-	[6]
Incidence ratio of EV71-associated herpangina to EV71-associated HFMD	0.069	0.044–0.11	[11,14,15]
Complication spectrum of severe EV71-associated HFMD			
Encephalitis only	41.3%	-	[18]
Aseptic meningitis only	7.4%	-	[18]
Acute flaccid paralysis only	0.5%	-	[18]
Acute flaccid paralysis combined with encephalitis	1.6%		[18]
Pulmonary oedema/hemorrhage only	12.1%	-	[18]
Pulmonary oedema/hemorrhage combined with encephalitis	33.7%		[18]
Myocarditis only	0.7%	-	[18]
Myocarditis combined with encephalitis	2.7%		[18]
Hospitalization rate of EV71-associated HFMD	16.8%	± 50%	[19,20]
Frequency of outpatient visit for EV71-associated HFMD and herpangina	1.8	-	[28–34]
Efficacy of vaccination against EV71-associated HFMD and herpangina			
Full dose	95%	90%-98%	[9–11]
One dose	50%	0–95%	Assumed
Rate of mild adverse events after vaccination	56%	47%-71%	[9–11]
Rate of serious adverse events after vaccination	0.04%	0–0.1%	[9–11]
Vaccine coverage			
First dose	95%	85%-100%	[22]
Second dose	93%	83%-100%	[22]
Disability weight			
Mild HFMD	0.056	-	[12,27]
Herpangina	0.056	-	[12,27]
Encephalitis	0.615	0.613–0.616	[12,27]
Aseptic meningitis	0.615	0.613–0.616	[12,27]
Acute flaccid paralysis	0.369	-	[12,27]
Pulmonary oedema/hemorrhage	0.252	0.201–0.300	[12,27]
Myocarditis	0.252	0.201–0.300	[12,27]
Duration of mild HFMD and herpangina	7 days	-	[25]
Duration of severe HFMD	16 days	16–32 days	[26]
Costs, US Dollars in 2012			
Outpatient visit due to EV71-associated HFMD, for each child	$163	± 50%	[28–34]
Outpatient visit due to EV71-associated herpangina, for each child	$52	± 50%	[28]
Hospitalization due to EV71-associated HFMD	$1,104	± 50%	[28–34]
Vaccine price, per dose	$2.5-$40		Assumed
Administration of vaccination	$0.5	± 50%	[35]
Mild adverse effects from vaccination	$1.75	± 50%	[12]
Serious adverse effects from vaccination	$29	± 50%	[36]
Discount rate	3%	0–10%	[38]

EV71: enterovirus 71; HFMD: hand, foot and mouth disease.

Herpangina has not been included in surveillance system in mainland China. The reporting of cases of herpangina and EV71 are aggregated together in Taiwan; specific data on the epidemiology of herpangina are not available. Fortunately, studies supplied information to calculate the ratio of patients with EV71-associated herpangina to that of EV71-associated HFMD. The ratio ranged from 0.044 to 0.11, with weighted mean of 0.069, using study sample size as the weight[11,14,15] (Table 1). The incidence of EV71-associated herpangina was calculated using this ratio and the incidence of EV71-associated HFMD from the China CDC[6].

### Complications of severe EV71-associated HFMD

Several studies reported the spectrum of complications of severe EV71-associated HFMD; nevertheless, they were single-center in design, had small-sized sample or short duration of enrollment, or the cases were selected[14,15,16,17]. Chen *et al* summarized hospitalized cases of EV71-associated HFMD in Taiwan from 1998 to 2005[18]. These cases were reported to surveillance systems at the Taiwan CDC by 538 hospitals of various levels. Based on them, the proportion of each complication was calculated for this analysis (Table 1).

### EV71-associated outpatient visits and hospitalizations

The data from the largest pediatric infectious disease center in Shanghai between 2007 and 2010 showed a hospitalization rate of 14% for all 28,058 patients diagnosed as HFMD and 54% of the inpatients were positive for EV71[19]. The hospitalization rate of EV71-associated HFMD was calculated as: 0.14×(0.54/0.45) = 0.168, in which 0.45 represented the proportion of EV71 in all HFMD cases according to the China CDC[6] (Table 1). Another survey from Guangdong reported a similar hospitalization rate [20].

The frequencies of outpatient visits for each symptomatic case were not available specifically for EV71-associated HFMD and herpangina. The frequency for overall HFMD patients was used in this analysis (Table 1).

### Efficacy, safety, schedule, and coverage of vaccination

Recently, three multicenter, randomized, double-blind, placebo-controlled phase 3 trials evaluated the efficacy and safety of inactivated EV71 vaccines in healthy infants and young children in China[9,10,11]. The 1-year efficacies ranged from 90% to 97.4% against EV71-associated HFMD. We performed a meta-analysis using a random-effect model. The results showed an overall efficacy of 95%, with 95% confidence interval of 90%-98%.

One of the trials reported efficacy against EV71-associated herpangina[11]. However, due to sparse events, no significant result was reached. In the absence of other data, this analysis assumed that the vaccine efficacy against EV71-associated herpangina was the same as that against HFMD.

Extended follow-up of one trial showed that the antibody titers were maintained at a high level through two years post-vaccination [9,21]. There are no long-term results for the other two trials. However, one of them reported consistent titers from month 6 to month 12 post-vaccination [11] and the other one reported slightly waned titers at day 180 after vaccination [10]. Therefore, we assumed that the titers do not wane significantly in our model, of which the time horizon is just five years.

The rate of adverse events within 28 days after vaccination was 56% on average (range 47%-71%)(Table 1)[9,10,11]. Most of the adverse events of EV71 vaccines were mild. Serious adverse events, which were considered to be associated or most likely associated with vaccination, happened only in 0.04% of the participants (range 0–0.1%)(Table 1)[9,10,11].

The schedule of vaccination against EV71 was two doses, 4 weeks apart[9,10,11], given at 3 and 4 months of age [22]. As this schedule is the same as that for the first two doses of diphtheria, tetanus and pertussis (DTP) vaccine, DTP coverage was used to estimate EV71 vaccine coverage[22]. Due to the lack of data on the coverage of the second DTP dose, data on the third DTP dose was used. Data are limited to estimate the efficacy of a single dose of EV71 vaccine. It was assumed to be 50% in this analysis[22].

### DALYs

We estimated DALYs using 2010 life expectancy data of China[23]. DALYs are the sum of years of life lost (YLLs) and years of life lost due to disability (YLDs)[24].

The durations of herpangina and mild HFMD are both 7 days on average[25]. According to Xu *et al*, the mean duration of hospitalization of severe HFMD (including critical cases) was 16 days[26]. There is no data on the duration of disability after discharge from hospital. Therefore, the duration was underestimated. In the base-case analysis, we assumed no disability following discharge. In sensitivity analysis, we explored how its uncertainty influenced the cost-effectiveness results.

Disability weights (DW) for each condition were taken from the World Health Organization’s estimates and a previous cost-effectiveness analysis (Table 1)[12,27]. DWs for conditions with combined complications were not available. For simplicity, the highest DW was used if the patients suffered more than one complication.

### Costs

This cost-effectiveness analysis was conducted from a societal perspective. The costs for EV71-associated HFMD and herpangina incorporated direct medical costs and non-medical costs for physician visits, medications, lab tests, and transportation, and indirect costs for work loss (S1 and S2 Tables). To today, seven studies have gathered these cost data from outpatient visits and hospitalizations in various regions of China[28,29,30,31,32,33,34]. The reported costs were weighted by the reported number of cases in each study to estimate average costs for each treatment setting (outpatient or hospitalization) (Table 1).

In China, vaccines are either supplied by commercial market or Expanded Program on Immunization (EPI). The latter is paid by the government. This analysis is to give an implication whether EV71 vaccines should be included in EPI in China. As the prices of vaccines in EPI are no more than $4.59 per dose, the analysis showed more concern for the case of $5.0 per dose (close to $4.59). As far as we know, recently EV71 vaccines have become commercially available in China and the price is around $30-$40 per dose, varied among regions. Right now the vaccines are paid by parents and the coverage is relatively low according to experiences from other commercial vaccines in China. The vaccine price may change in the future. Therefore, we performed the analysis at a range of prices for vaccines. Our analysis used the range from $2.5 to $40 ($2.5, $5, $10, $20, $30, $40) per dose because this range covers almost all prices of China made vaccines.

The price of vaccine administration was estimated at 3 Chinese Yuan (CNY) per injection according to subsidy policies to health facilities for vaccine administration (range 2–4 CNY, to cover the costs of nurse labor, syringe and transportation and storage of vaccine)[35]. The costs of vaccine-associated adverse events were considered in this analysis and they were obtained from published studies (Table 1)[12,36]. All costs were converted to 2012 US Dollars (1 US Dollar = 6.30 CNY) using the medical care component of the Consumer Price Index[37].

### Cost-effectiveness analysis

Incremental cost-effectiveness ratio (ICER) was calculated using the following formula:

$$\text{ICER}=\left({\text{Cost}}_{\text{no}}{\;}_{\text{vaccination}}{-\text{Cost}}_{\text{vaccination}}\right)\text{/}\left({\text{Effect}}_{\text{no}}{}_{\text{ vaccination}}{-\text{Effect}}_{\text{vaccination}}\right)$$

The numerator was the difference in total costs with or without vaccination. The denominator was DALYs that vaccination averted. There is no official guidance on discounting in China. All costs and DALYs were discounted to 2012 amounts at a rate of 3% annually (range 0–10%) according to Weinstein *et al*[38]. The cost-effectiveness thresholds were based on the WHO standard (highly cost-effective, ICER < GDP per capita; cost-effective, GDP per capita < ICER < 3×GDP per capita; and not cost-effective, ICER > 3×GDP per capita)[39]. The GDP per capita for China in 2012 was approximately $6,300[37].

### Sensitivity analysis

To assess the robustness of the model and to identify influential model inputs for which additional data are warranted, one-way sensitivity analyses were performed at each level of vaccine price. There are substantial heterogeneities of disease incidence and costs among different regions in China. Thus, a two-way sensitivity analysis was performed to evaluate their influence on the base-case results for the case of $5 per dose. The ranges of model inputs for sensitivity analysis were all listed in Table 1. All data used in this study are available through references.

## Results

### Base-case analysis

Table 2 shows the clinical events in a birth cohort of 1,000,000 Chinese infants followed for five years with or without EV71 vaccination. EV71 vaccination would be expected to annually avert 37,872 cases of EV71-associated HFMD, 2,629 cases of EV71-associated herpangina, 72,900 outpatient visits, 6,363 admissions to hospital, 29 deaths, and 945 DALYs among children younger than five years.

**Table 2:** Expected health outcomes related to enterovirus 71 (EV71) in a birth cohort of 1,000,000 Chinese infants followed for five years with or without EV71 vaccination.

Outcome	No vaccination	Vaccination	Averted events
Mild HFMD cases	41,401	4,409	36,992
Severe HFMD cases	952	101	851
Deaths	32	3	29
Herpangina cases	2,942	313	2,629
Outpatient visits	81,589	8,689	72,900
Hospitalizations	7,121	758	6,363
YLLs	1,040	111	929
YLDs	18	2	16
DALYs	1,058	113	945

DALYs: disability adjusted life years; HFMD: hand, foot and mouth disease; YLDs: years of life lost due to disability; YLLs: years of life lost.

The economic burden of EV71-associated HFMD and herpangina incorporating direct and indirect costs is approximately 13 million dollars per 1,000,000 Chinese infants followed for five years. Table 3 shows the costs per DALY averted by the EV71 vaccination program at various prices per dose. According to WHO cost-effectiveness criteria, the vaccination program would be cost-saving at $2.5 and $5.0 per dose, cost-effective at $10, and not cost-effective at $20, $30 and $40. The break-even price of the vaccine is $5.2 per dose. When the price is less than $8.3 or $14.6 per dose, the vaccination program would be highly cost-effective or cost-effective, respectively.

**Table 3:** Base-case cost-effectiveness results in a birth cohort of 1,000,000 Chinese infants followed for five years with or without EV71 vaccination (US dollars in 2012).

Price of per vaccine dose, $	Total cost of vaccination, $	Net cost of vaccination, $	ICER cost per DALY averted, $
2.5	7,966,122	-5,029,962	Cost-saving
5.0	12,666,122	-329,962	Cost-saving
5.2	12,996,084	0	0 (Break-even)
8.3	18,951,338	5,955,254	6,300 (GDP per capita)
10	22,066,122	9,070,038	9,595
14.6	3,0861,845	17,865,761	18,900 (3×GDP per capita)
20	40,866,122	27,870,038	29,483
30	59,666,122	56,670,038	49,372
40	78,466,122	65,470,038	69,260

The GDP per capita for China in 2012 was approximately $6,300.

DALY: disability adjusted life years; ICER: incremental cost-effectiveness ratio.

### Sensitivity analysis

**Fig 2:**
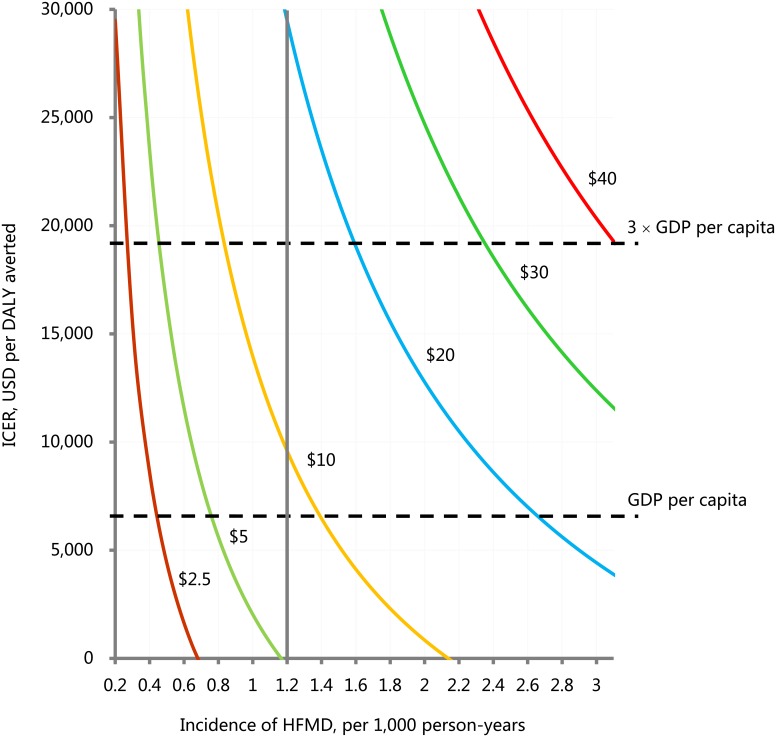
The relationship of hand, mouth and foot disease incidence on the incremental cost-effectiveness ratio of EV71 vaccination comparing with no EV71 vaccination. Each curve represents this relationship at a certain vaccine price per dose. The overall incidence of HFMD was 1.2 per 1,000 person-years from 2008 to 2012 in China. The gross domestic product (GDP) per capita for China in 2012 was approximately $6,300. 3×GDP per capita was approximately $18,900. DALY: disability adjusted life year; HFMD: hand, mouth and foot disease; ICER: incremental cost-effectiveness ratio; USD: US dollars.

Fig 2 shows the impact of HFMD incidence on the cost-effectiveness of EV71 vaccination at various prices per dose. As the incidence of HFMD falls below 0.3, 0.5, 0.9, 1.6, 2.4, and 3.2 per 1,000 person-years, the vaccination program would be not cost-effective at the prices per dose of $2.5, $5, $10, $20, $30, and $40, respectively.

Table 4 shows how other parameters influence the ICER comparing EV71 vaccination with no vaccination. At prices per dose less than $5, EV71 vaccination is still cost-saving or highly cost-effective when the parameters are varied across their ranges. At $10, the discount rate is the only influential parameter. When the cost and DALYs are both discounted at a rate more than 6%, EV71 vaccination would be no longer cost-effective. At $20, EV71 vaccination would be cost-effective only when the cost and DALYs are both discounted at a rate less than 2%. At the prices more than $30, EV71 vaccination would not be cost-effective when any parameter in Table 4 is varied across its range. A series of tornado diagrams show the rank of parameters’ influence on ICER at the prices per dose of $10, $20, $30, and $40, respectively (S1 Fig).

**Table 4:** One-way sensitivity analyses comparing EV71 vaccination with no EV71 vaccination at various prices per dose (US Dollars in 2012).

$2.5	$5.0	$10 (9,595)^§^	$20 (29,483) ^§^	$30 (49,372) ^§^	$40 (69,260) ^§^
Incidence ratio of EV71-associated herpangina to EV71-associated HFMD, 0.044–0.11					
Cost-saving	Cost-saving	9,509 ~ 9,647 (-1% ~ 1%)	29,388 ~ 29,541 (0% ~ 0%)	49,267 ~ 49,435 (0% ~ 0%)	69,146 ~ 69,329 (0% ~ 0%)
Hospitalization rate of EV71-associated HFMD, ±50%					
Cost-saving	Cost-saving ~ 264	6,605 ~ 12,586 (-31% ~ 31%)	26,493 ~ 32,474 (-10% ~ 10%)	46,381 ~ 52,362 (-6% ~ 6%)	66,270 ~ 72,251 (-4% ~ 4%)
Efficacy of vaccination against EV71-associated HFMD and herpangina (full dose 90%-98%, one dose 0–95%)^‡^					
Cost-saving	Cost-saving ~ 457	8,727 ~ 11,072 (9% ~ 15%)	27,827 ~ 32,303 (6% ~ 10%)	46,926 ~ 53,534 (5% ~ 8%)	66,026 ~ 74,765 (5% ~ 8%)
Rate of mild adverse events after vaccination, 47%-71%					
Cost-saving	Cost-saving	9,437 ~ 9,859 (-2% ~ 3%)	29,325 ~ 29,747 (-1% ~ 1%)	49,213 ~ 49,636 (0% ~ 1%)	69,102 ~ 69,524 (0% ~ 0%)
Rate of serious adverse events after vaccination, 0–0.1%					
Cost-saving	Cost-saving	9,583 ~ 9,613 (0% ~ 0%)	29,472 ~ 29,501 (0% ~ 0%)	49,360 ~ 49,389 (0% ~ 0%)	69,248 ~ 69,278 (0% ~ 0%)
Vaccine coverage (first dose 85%-100%, second dose 83%-100%)^‡^					
Cost-saving	Cost-saving	9,595 ~ 9,597 (0% ~ 0%)	29,482 ~ 29,496 (0% ~ 0%)	49,370 ~ 49,396 (0% ~ 0%)	69,256 ~ 69,295 (0% ~ 0%)
Disability weight (encephalitis 0.613–0.616, aseptic meningitis 0.613–0.616) ^‡^					
Cost-saving	Cost-saving	9,595 ~ 9,595 (0% ~ 0%)	29,483 ~ 29,484 (0% ~ 0%)	49,371 ~ 49,373 (0% ~ 0%)	69,260 ~ 69,261 (0% ~ 0%)
Disability weight (pulmonary oedema/hemorrhage 0.201–0.300, myocarditis 0.201–0.300) ^‡^					
Cost-saving	Cost-saving	9,595 ~ 9,596 (0% ~ 0%)	29,482 ~ 29,485 (0% ~ 0%)	49,369 ~ 49,375 (0% ~ 0%)	69,256 ~ 69,264 (0% ~ 0%)
Duration of severe HFMD, 16–32 days					
Cost-saving	Cost-saving	9,546 ~ 9,595 (-1% ~ 0%)	29,331 ~ 29,483 (-1% ~ 0%)	49,117 ~ 49,372 (-1% ~ 0%)	68,903 ~ 69,260 (-1% ~ 0%)
Cost of mild adverse events after vaccination, ±50%					
Cost-saving	Cost-saving ~ 143	9,103 ~ 10,088 (-5% ~ 5%)	28,991 ~ 29,976 (-2% ~ 2%)	48,879 ~ 49,864 (-1% ~ 1%)	68,768 ~ 69,753 (-1% ~ 1%)
Cost of serious adverse events after vaccination, ±50%					
Cost-saving	Cost-saving	9,589 ~ 9,601 (0% ~ 0%)	29,478 ~ 29,489 (0% ~ 0%)	49,366 ~ 49,378 (0% ~ 0%)	69,254 ~ 69,266 (0% ~ 0%)
Cost of outpatient visit due to EV71-associated HFMD, ±50%					
Cost-saving	Cost-saving ~ 2,216	7,030 ~ 12,160 (-27% ~ 27%)	26,918 ~ 32,049 (-9% ~ 9%)	46,806 ~ 51,937 (-5% ~ 5%)	66,695 ~ 71,825 (-4% ~ 4%)
Cost of outpatient visit due to EV71-associated herpangina, ±50%					
Cost-saving	Cost-saving	9,527 ~ 9,663 (-1% ~ 1%)	29,415 ~ 29,552 (0% ~ 0%)	49,303 ~ 49,440 (0% ~ 0%)	69,192 ~ 69,328 (0% ~ 0%)
Cost of hospitalization due to EV71-associated HFMD, ±50%					
Cost-saving	Cost-saving ~ 3,159	6,087 ~ 13,104 (-37% ~ 37%)	25,975 ~ 32,992 (-12% ~ 12%)	45,863 ~ 52,880 (-7% ~ 7%)	65,752 ~ 72,769 (-5% ~ 5%)
Cost of vaccine administration, ±50%					
Cost-saving	Cost-saving ~ 148	9,098 ~ 10,092 (-5% ~ 5%)	28,986 ~ 29,981 (-2% ~ 2%)	48,875 ~ 49,869 (-1% ~ 1%)	68,763 ~ 69,757 (-1% ~ 1%)
Discount rate for both cost and DALYs, 0–10%					
Cost-saving	Cost-saving ~ 4,257	3,520 ~ 42,004 (-63% ~ 338%)	11,411 ~ 117,500 (-61% ~ 299%)	19,303 ~ 192,995 (-61% ~ 291%)	27,195 ~ 268,491 (-61% ~ 288%)
Discount rate for cost only, 0–10%					
Cost-saving	Cost-saving ~ 1,121	8,871 ~ 11,065 (-8% ~ 15%)	28,759 ~ 30,954 (-2% ~ 5%)	48,647 ~ 50,842 (-1% ~ 3%)	68,536 ~70,730 (-1% ~ 2%)

The percentages in parentheses represent the changes of incremental cost-effectiveness ratios from base-case analyses. The GDP per capita for China in 2012 was approximately $6,300.

DALYs: disability adjusted life years; EV71: enterovirus 71; HFMD: hand, foot and mouth disease.

^**§**^The numbers in parentheses represent the incremental cost-effectiveness ratios in base-case analyses.

^‡^The parameters in parentheses simultaneously change over their own value ranges.

**Fig 3:**
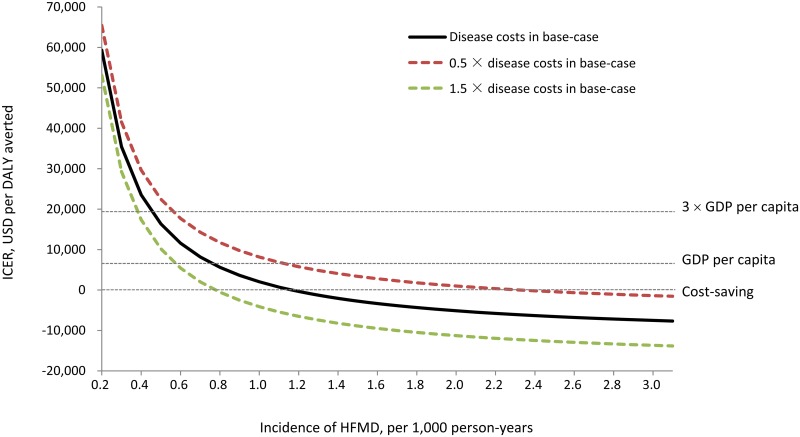
The influence of hand, mouth and foot disease incidence and disease costs on the incremental cost-effectiveness ratio of EV71 vaccination comparing with no EV71 vaccination. Disease costs include costs of outpatient visits and hospitalizations due to EV71 infections. The gross domestic product (GDP) per capita for China in 2012 was approximately $6,300. 3×GDP per capita was approximately $18,900. DALY: disability adjusted life year; HFMD: hand, mouth and foot disease; ICER: incremental cost-effectiveness ratio; USD: US dollars.

Fig 3 shows how HFMD incidence and disease costs influence the ICER when the vaccine price is $5 per dose. If the disease costs increase by 50%, the vaccination program would be not cost-effective in regions where the incidence of HFMD is below 0.4 per 1,000 person-years. If the disease costs decrease by 50%, the incidence making vaccination not cost-effective is below 0.6 per 1,000 person-years.

## Discussion

The results of this study suggest that a national vaccination program against EV71 would result in substantial decline in morbidity, mortality, use of health service, and DALYs in China. Based on an actual Chinese birth cohort size of around 15 million a year[40], EV71 vaccination would be expected to annually avert 567,500 cases of EV71-associated HFMD, 40,000 cases of EV71-associated herpangina, 1,093,500 outpatient visits, 95,500 admissions to hospital, 435 deaths, and 14,000 DALYs among children younger than five years. As EV71-associated early death contributes to the great majority of DALYs (Table 2) and current vaccines are highly effective, health benefit of the program mainly comes from its role in avoiding EV71-associated death.

The prices of vaccines in China’s routine childhood immunization program paid by the government are no more than $4.59 per dose[22]. Therefore, if the vaccine is priced in accordance with previous prices of under $4.59, then this vaccine would be cost-saving. This result is only sensitive to the HFMD incidence among all clinical and cost parameters. If the HFMD incidence is below than 0.5 per 1,000 person-years, that is, the incidence of EV71-associated HFMD is below 0.23 per 1,000 person-years, a national EV71 vaccination program would be unaffordable at the price per dose of $5. If the program is determined by local government, Western China provinces (Tibet, Xinjiang, Sichuan, Qinghai, and Gansu) with the lowest incidences should carefully balance the program’s economic cost and health benefit[13]. Besides the HFMD incidence, the discount rate, costs of EV71-associated HFMD, and hospitalization rate due to EV71-associated HFMD are the most influential parameters on the ICER comparing vaccination with no vaccination. However, at the price of $5 per dose and with the baseline incidence, they do not affect the cost-effectiveness.

This study has several methodological strengths. First, three large RCTs of high quality supplied solid clinical evidence on the efficacy and safety of EV71 vaccines in Chinese infants and children[9,10,11]. Second, nationwide epidemiology data on EV71-associated diseases were available and clinical outcomes were based on large populations[6,18]. Besides, the incidences of EV71-associated HFMD were age-specific so that we could assess the disease burden more precisely. Third, the disease costs incorporating direct and indirect costs came from seven surveys across regions varied in economic development levels in China[28,29,30,31,32,33,34]. These cost data benefited our study.

Due to lack of data, our study has several limitations. First, we did not consider long-term sequelae in EV71-infected children with severe complications. Although most recovered, two studies showed that some children with EV71 brainstem encephalitis (especially stage III) had residual cognitive and motor deficits at follow-ups after their hospitalization[41,42]. Second, we did not consider EV71-associated diseases other than HFMD and herpangina, mainly including upper respiratory tract infection and diarrhea. Third, according to the experience of our center and other pediatric centers in China, some children with severe complications died after discharge and they are not reported to the disease surveillance system. Fourth, this study adopted a static cohort model instead of a dynamic infection transmission model because the vaccine’s impact on the overall force of infection is not clear. Thus, the current analysis underestimated the disease burden of EV71 infection as well as vaccine effect. If the above four factors are considered, vaccination program would be more cost-effective.

Before the emergence of clinical evidence on EV71 vaccines, a previous study forecasted the economic value of a future vaccine against EV71[12]. Although the study also prefers routine vaccination in China (cost-effective when vaccine cost is $25 and efficacy ≥70% or cost is $10 and efficacy ≥50%), several key clinical and cost parameters are different between the study and ours: (1) based on 2009 data, the incidence of EV71 infection was lower than ours which was based on data of four years; (2) a percentage of more than 26% of severe cases in all EV71 infected cases was quite high compared to our data from the China CDC; (3) the efficacy of vaccine ranged from 50%-90%, which was quite lower than ours (90%-98%); (4) disease costs came from an American population and was converted to China hospital costs using an indirect method. These costs were much lower than those from recent seven studies directly surveying economic burden of the disease[28,29,30,31,32,33,34].

The EV71 vaccines in the three phase 3 trials were all developed on the basis of subgenotype strain C4. Fortunately, other subgenotype strains of EV71 have not been reported in mainland China [8,43]. However, in other Asian regions, B4, B5, C2, and C5 have been reported[8,43,44]. Studies showed that C4 vaccine could elicit cross-neutralizing response with other subgenotype strains[45,46,47]. Nevertheless, the degree of cross-protective immunity and the potential escape evolution for EV71 are unknown. More importantly, this cross-protective immunity has not been tested in clinical trials. The incidence of EV71-associated diseases, disease costs, and medical resources in other Asian regions are all different from China. Further studies should evaluate the public health impact and economic value of these vaccines in other Asian regions.

This study focused on EV71 vaccination versus no EV71 vaccination. Future analyses should evaluating the cost-effectiveness of vaccination against Coxsackievirus A 16 (CA16) or other enteroviruses causing HFMD. The predominance of CA16 is comparable with EV71 nationwide and CA16 often becomes the main epidemic strain in some regions of China[6]. Strategies of EV71 or CA16 vaccination alone and combined vaccination may need evaluation. Chen *et al* have shown that the co-administration of inactivated EV71 vaccine with a commercial pentavalent vaccine did not affect the antibody response of each vaccine[48]. When considering the incorporation of EV71 vaccine into the EPI, future analyses should also evaluate strategies of co-administration with other EPI vaccines.

### Conclusions

A national EV71 vaccination program would prevent a substantial portion of EV71 related morbidity, mortality, outpatient visits, and admissions to hospitals among children younger than five years in China. Within the price range of current routine vaccines paid by the government, the program is cost-saving or highly cost-effective. Policy makers should consider including EV71 vaccination as part of China’s routine childhood immunization schedule.

## Supporting information

S1 Table Cost components for outpatient visit. (DOC)

S2 Table Cost components for hospitalization. (DOC)

S1 Fig Tornado diagrams of one-way sensitivity analyses. (PDF)
