# Supplementary material for: Cost-effectiveness of a national enterovirus 71 vaccination program in China
Source: PLoS Negl Trop Dis. 2017 Sep 11;11(9):e0005899. doi: 10.1371/journal.pntd.0005899 (PMC5608421; doi:10.1371/journal.pntd.0005899)
Supplement: S1 Table — (DOC) [file pntd.0005899.s001.doc]

S1 Table. Cost components for outpatient visit

| Costs for outpatient visit | Direct medical costs | Medications |
| --- | --- | --- |
|  |  | Lab tests |
|  |  | Physician visit |
|  |  | Home care |
|  | Direct non-medical costs | Transportation |
|  |  | Communication |
|  |  | Dietary changes |
|  |  | Other costs |
|  | Indirect costs | Work loss incurred by parents caring for their sick child |
