# Supplementary material for: Cost-effectiveness of a national enterovirus 71 vaccination program in China
Source: PLoS Negl Trop Dis. 2017 Sep 11;11(9):e0005899. doi: 10.1371/journal.pntd.0005899 (PMC5608421; doi:10.1371/journal.pntd.0005899)
Supplement: S2 Table — (DOC) [file pntd.0005899.s002.doc]

S2 Table. Cost components for hospitalization

| Costs for hospitalization | Direct medical costs | Medications and other treatments |
| --- | --- | --- |
|  |  | Lab tests |
|  |  | Other examinations (e.g. X-ray) |
|  |  | Physician care |
|  |  | Nursing care |
|  |  | Bed charges |
|  | Direct non-medical costs | Transportation |
|  |  | Communication |
|  |  | Dietary changes |
|  |  | Other costs |
|  | Indirect costs | Work loss incurred by parents caring for their sick child |
