# Supplementary material for: Cost-effectiveness of a national enterovirus 71 vaccination program in China
Source: PLoS Negl Trop Dis. 2017 Sep 11;11(9):e0005899. doi: 10.1371/journal.pntd.0005899 (PMC5608421; doi:10.1371/journal.pntd.0005899)
Supplement: S1 Fig — (PDF) [file pntd.0005899.s003.pdf]

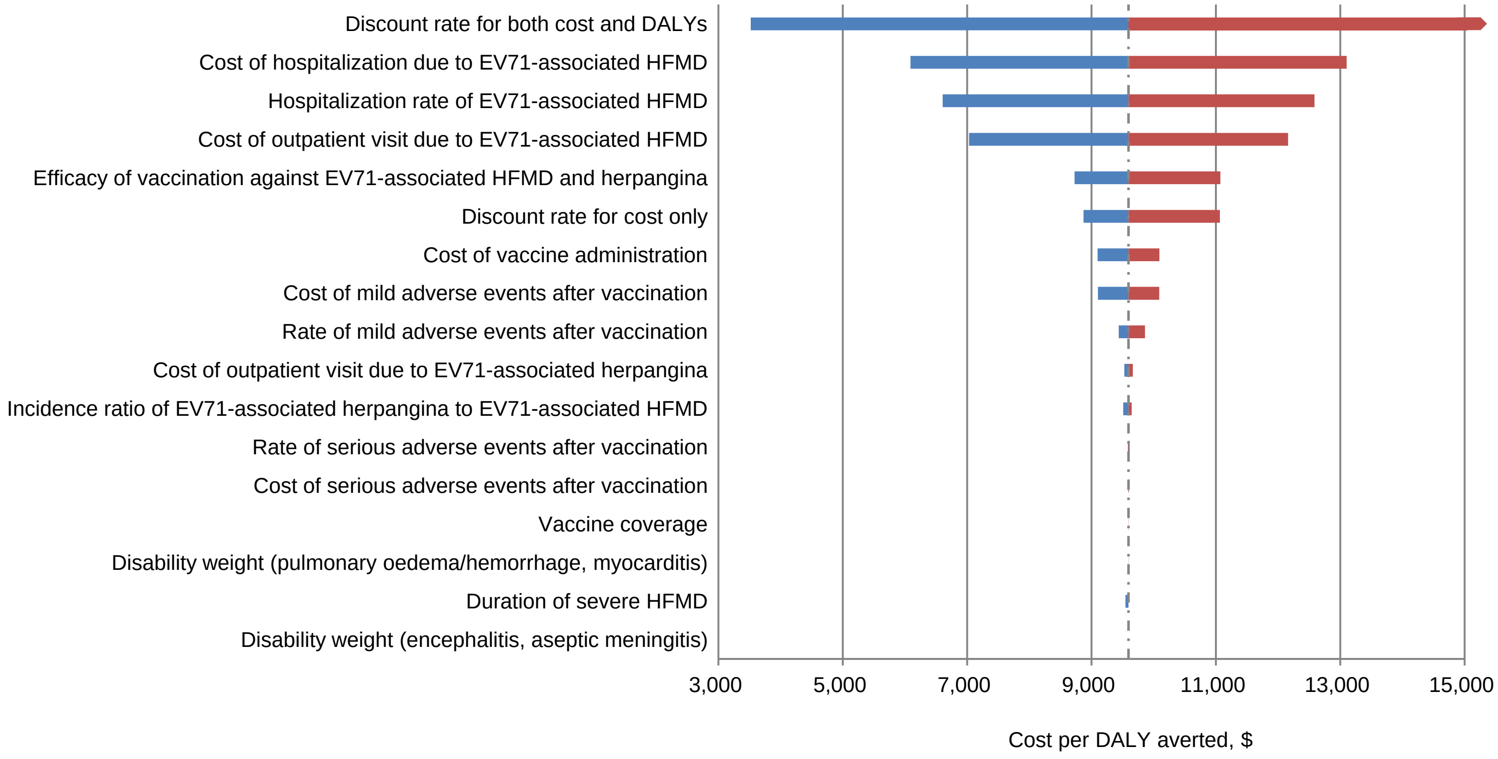

Tornado diagram of univariate sensitivity analyses for EV71 vaccination versus no vaccination at the price per dose of $10. HFMD: hand, foot and mouth disease; EV71: enterovirus 71; DALYs: disability adjusted life years.

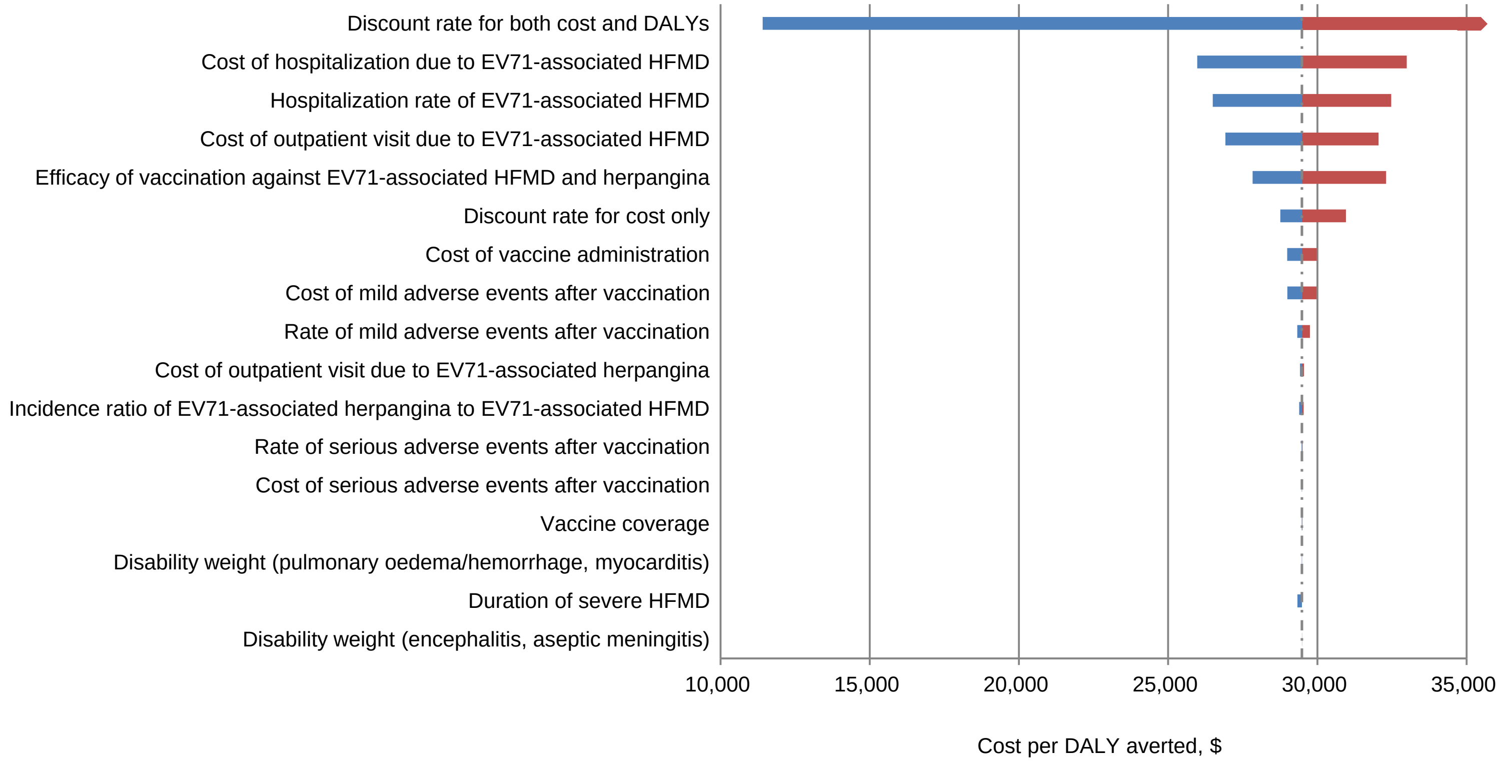

Tornado diagram of univariate sensitivity analyses for EV71 vaccination versus no vaccination at the price per dose of $20.
HFMD: hand, foot and mouth disease; EV71: enterovirus 71; DALYs: disability adjusted life years.

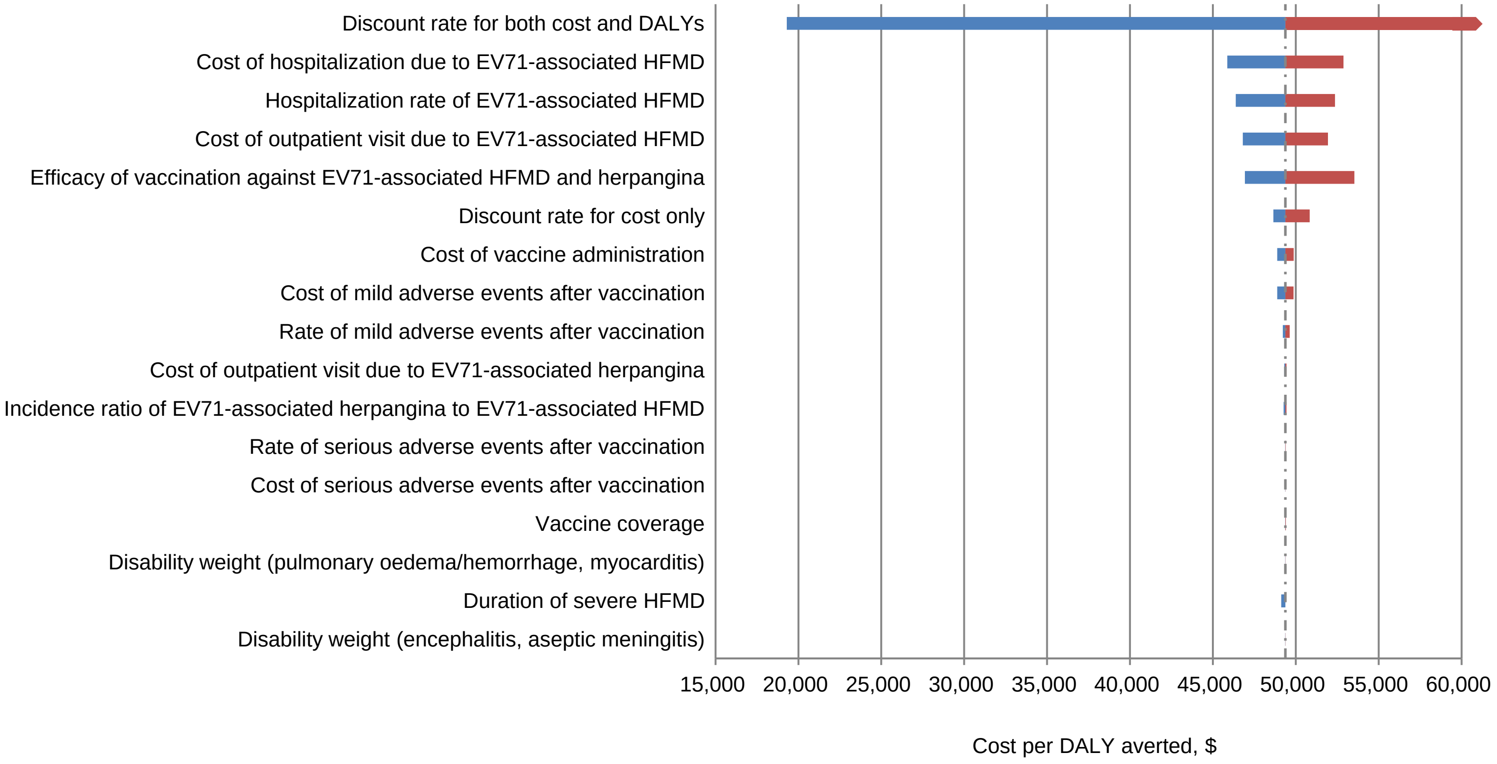

Tornado diagram of univariate sensitivity analyses for EV71 vaccination versus no vaccination at the price per dose of $30. HFMD: hand, foot and mouth disease; EV71: enterovirus 71; DALYs: disability adjusted life years.

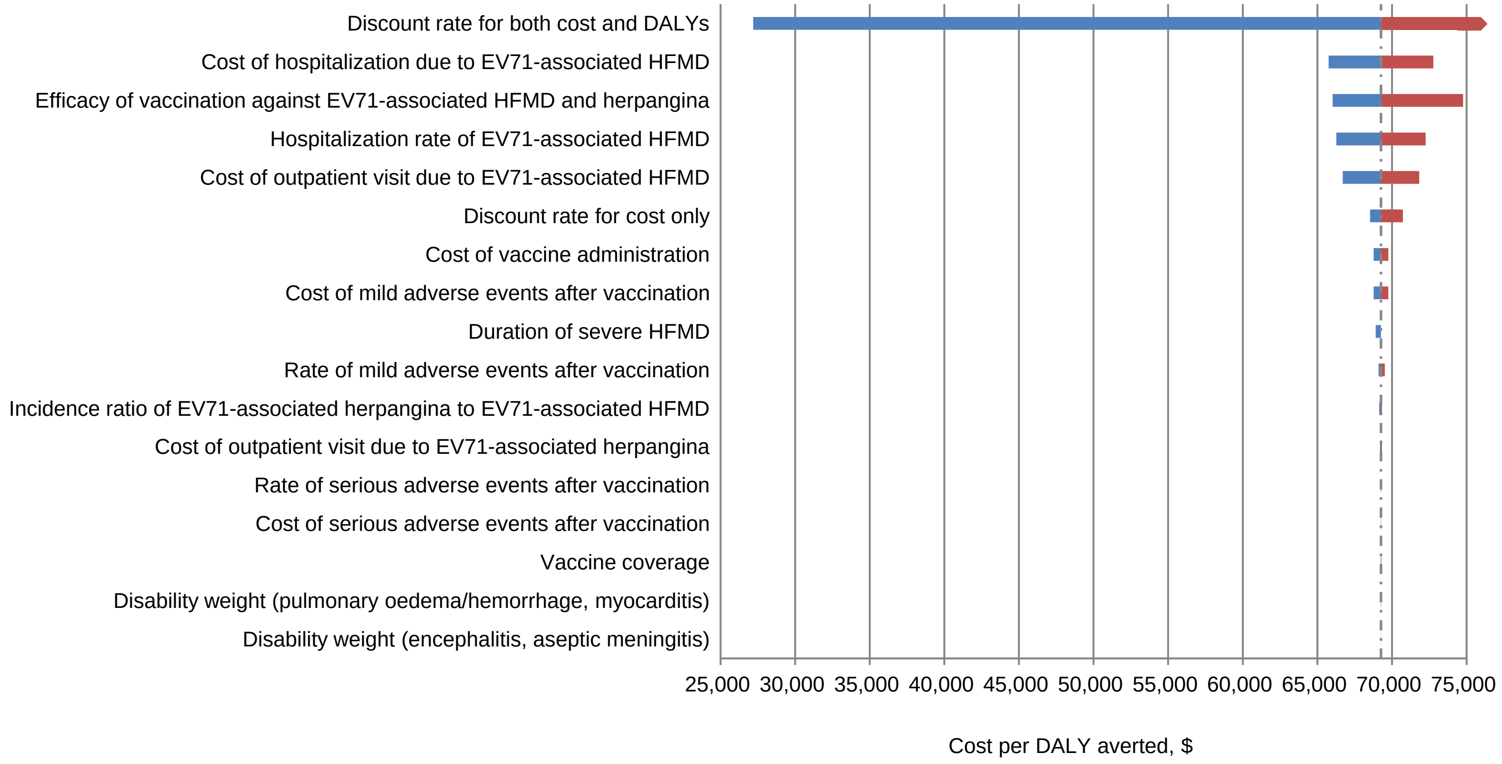

Tornado diagram of univariate sensitivity analyses for EV71 vaccination versus no vaccination at the price per dose of $40.
HFMD: hand, foot and mouth disease; EV71: enterovirus 71; DALYs: disability adjusted life years.
